# NUDT21 Regulates Macrophage Cytokine Responses via Alternative Polyadenylation in ARDS

**DOI:** 10.21203/rs.3.rs-7810883/v1

**Published:** 2025-10-24

**Authors:** Tingting Mills, Hui Liu, Yu Wang, Scott Collum, Kai-Lieh Huang, Maria Gacha-Garay, Sarah Shin, Xiaoyi Yuan, Megan Ballinger, Hari Yalamanchili, Eric Wagner, Harry Karmouty

**Affiliations:** University of Texas Health Science Center at Houston; University of Texas Health Science Center at Houston; University of Texas Health Science Center at Houston; UTHealth Houston; University of Rochester; Stony Brook University; New York University Langone Medical Center; The University of Texas Health Science Center Houston; The Ohio State University Wexner Medical Center; Baylor College of Medicine; University of Rochester; UTHealth houston

**Keywords:** alternative polyadenylation, macrophage, NUDT21, Acute respiratory distress syndrome

## Abstract

Acute respiratory distress syndrome (ARDS) is a life-threatening condition driven by uncontrolled inflammation and immune dysregulation. The post-transcriptional mechanisms that fine-tune macrophage activation in ARDS remain poorly understood. Here, we identify Nudix hydrolase 21 (NUDT21) as a critical regulator of macrophage-mediated inflammation through alternative polyadenylation (APA). NUDT21 was downregulated in macrophages from human and mouse ARDS lungs. Functional studies using macrophage-specific Nudt21 knockout mice (Nudt21^f/f^LysmCre, Nudt21^f/f^Cxcr1Cre, or mice with bone marrow transplantation) revealed that Nudt21 loss amplifies cytokine production, neutrophil infiltration, and lung injury in lipopolysaccharide or bleomycin-induced lung injury models. Notably, neutrophil depletion did not alleviate the exaggerated inflammation in Nudt21^f/f^LysmCre mice, confirming a macrophage-specific mechanism. In contrast, Nudt21^f/f^CD68 rtTA/tetOCre mice did not exhibit increased injury, likely because alveolar macrophages—but not recruited macrophages—play a major role in the LPS model. Transcriptome profiling revealed widespread 3’UTR shortening of inflammatory genes and elevated protein expression in NUDT21-deficient macrophages, indicating APA-mediated translational activation. Furthermore, we identified hypoxia-induced microRNA-181a as an upstream repressor of NUDT21, linking oxygen stress to APA remodeling. Collectively, these findings uncover a previously unrecognized hypoxia–miR-181a–NUDT21–APA axis that amplifies macrophage inflammation and lung injury.

## INTRODUCTION

Acute Respiratory Distress Syndrome (ARDS) is a common life-threatening respiratory failure that is characterized by the acute onset of noncardiogenic pulmonary edema, hypoxemia, and massive inflammation^1, 2, 3^. It is a severe complication of various conditions, including sepsis, pneumonia, trauma, and viral infections, leading to alveolar-capillary barrier dysfunction, excessive immune activation, and impaired gas exchange. Due to its rapid progression and high severity, ARDS accounts for approximately 10% of admissions to intensive care units^2^. Despite advances in supportive strategies such as mechanical ventilation and prone positioning, no effective pharmacological therapy exists, and mortality remains as high as 30–40%^4, 5, 6^. A major challenge in ARDS treatment is uncontrolled lung inflammation, and a central component of the inflammatory response in ARDS is the macrophages. Macrophages are innate immune cells that play critical roles in pathogen recognition, inflammation initiation and resolution, and tissue repair^7, 8, 9^. Tissue-resident alveolar macrophages (TR-AMs) are embryonically derived and essential for lung development and surfactant homeostasis^10, 11^. They serve as the first line of defense against pathogens and also facilitate the resolution of inflammation by clearing apoptotic cells. In contrast, monocyte-derived macrophages (MoMs) are recruited to the alveolar space in response to injury, where they exhibit a proinflammatory phenotype and release cytokines such as interleukin 1β (IL1B), IL6, IL8, C-C motif chemokine ligand 2 (CCL2), and tumor necrosis factor alpha (TNFA)^12, 13, 14^. Interstitial macrophages (IMs), located within the bronchial and alveolar interstitium, share a proinflammatory profile with MoMs but also rapidly upregulate anti-inflammatory cytokines to facilitate tissue repair^15, 16, 17^. While transcriptional control of macrophage activation has been extensively studied, how post-transcriptional mechanisms fine-tune these inflammatory responses in ARDS remains largely unknown.

Alternative polyadenylation (APA) represents a major yet underexplored layer of post-transcriptional control that shapes gene expression by selecting distinct polyadenylation sites (PAS) within the same transcript^18, 19^. Most mammalian genes, including key proinflammatory mediators, contain multiple polyadenylation sites (PAS)^18, 20^. The use of proximal PAS shortens the 3’UTR, often eliminating microRNA or RNA-binding protein recognition elements, thereby may enhance mRNA stability and translation. Several studies suggest a potential role for APA in macrophage activation and immune responses. An early study reported induction of cleavage stimulatory factor 64 (CstF64) and APA of a few genes in LPS-treated RAW 264.7 macrophages ^21^, suggesting the involvement of APA in macrophage activation. More recently, a genome-wide APA profiling study in macrophages infected with vesicular stomatitis virus (VSV) identified widespread 3’ UTR shortening in genes involved in multiple immune response pathways ^22^. Additionally, a systemic screening of genes in the lungs of COVID-19 patients identified APA genes involved in neutrophil activation. The authors suggested that these APA genes may be proinflammatory factors released from macrophages^23^. These findings provide a strong foundation for studying APA regulation in macrophages during ARDS. However, a functional investigation using animal ARDS models or protein-level validation of APA-regulated genes in ARDS remains necessary to establish its significance.

One key regulator of APA is Nudix Hydrolase 21 (NUDT21), a critical component of cleavage factor Im (CFIm). Our group and others have identified NUDT21 as a master regulator of APA, where its downregulation leads to 3’ UTR shortening and increased expression of genes involved in fibrosis^24, 25^ and cancer proliferation^26^. Given the central role of macrophages in ARDS and the sensitivity of APA to inflammatory stress, we hypothesized that loss of NUDT21 in macrophages amplifies cytokine production and lung injury through APA reprogramming. Here, we identify NUDT21 as a critical regulator of macrophage inflammatory responses in ARDS. Using several macrophage/myeloid-specific Nudt21 knockout mice, we demonstrate that NUDT21 loss enhances inflammatory cytokine expression, promotes 3’UTR shortening of key proinflammtory genes, and exacerbates lung injury. We further uncover that hypoxia-induced miR-181a represses NUDT21, linking oxygen stress to APA remodeling. These findings establish NUDT21-mediated APA as a fundamental mechanism controlling macrophage activation and inflammatory injury in ARDS.

## RESULTS

### NUDT21 is downregulated in macrophages in the lungs of human ARDS.

**Figure 1.**
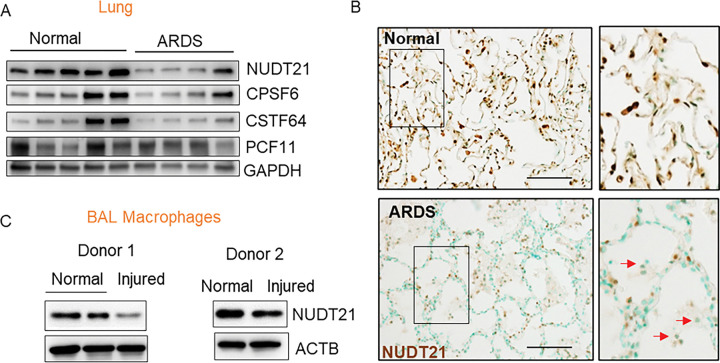
NUDT21 expression in human ARDS. (A) Western blot showing NUDT21, and other 3’UTR proteins CPSF6, CSTF64, and PCF11 levels in the lungs of normal donors and ARDS patients. (B) IHC of NUDT21 (brown) in normal and ARDS lungs. Arrow: immune cells. (C) NUDT21 protein expression in primary macrophages isolated from normal or injured lobes of donor C1070 and C1071. Scale bar=200μm.

Given the critical role of macrophage-mediated inflammation in ARDS and the emerging importance of APA in immune regulation, we investigated whether APA is implicated in human ARDS. For this purpose, we first examined the expression of NUDT21, a master APA regulator, along with key polyadenylation proteins known to influence APA, including Cleavage and Polyadenylation Specificity Factor 6 (CPSF6), Cleavage Stimulation Factor 64 kDa subunit (CSTF64), and Cleavage and Polyadenylation Factor Subunit (PCF11)^26, 27, 28^. While CPSF6 and CSTF64 showed a trend toward downregulation and PCF11 levels remained unchanged in ARDS lungs, NUDT21 consistently exhibited the most significant downregulation (Fig. 1A). Therefore, we will focus on NUDT21 for further study. Immunohistochemistry (IHC) revealed decreased NUDT21 expression in both lung epithelial cells and immune cells (Fig. 1B, arrows). Given prior evidence linking APA to macrophage responses in LPS- and virus-treated models^21, 22^, we next assessed NUDT21 expression in macrophages isolated from two donors who had different degrees of lung injury in different lobes. Interestingly, NUDT21 was downregulated in macrophages from injured lung lobes compared to those from unaffected lobes in both donors (Fig. 1C). Collectively, these data indicate that NUDT21 is downregulated in macrophages in human ARDS lungs, suggesting a potential role in ARDS pathogenesis.

### NUDT21 is downregulated in macrophages in murine models of ARDS.

**Figure 2.**
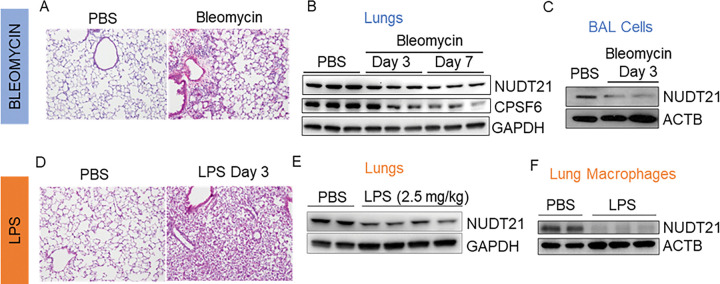
NUDT21 expression in mouse ARDS. (A) H&E staining of day 3 lungs of mice injected PBS or bleomycin (2.5U/kg) via oropharyngeal aspiration (OPA). (B-C) NUDT21 protein expression in the (B) lungs and (C) BAL cells from PBS or bleomycin injected mice. (D) H&E staining of day 3 lungs of mice injected with PBS or LPS (2.5 mg/kg bodyweight) via OPA. (D-F) NUDT21 expression in the (E) lungs and (F) primary isolated lung macrophages from day 3 mice treated with PBS or LPS.

Building on our findings in human ARDS, we next sought to determine whether NUDT21 downregulation also occurs in murine models of ARDS. We first assessed NUDT21 protein levels in the lungs of mice treated with either bleomycin (Fig. 2A) or lipopolysaccharide (LPS) via oropharyngeal aspiration (OPA) (Fig. 2D). Consistent with our human data, NUDT21 was downregulated in the lungs of bleomycin-treated mice as early as day 3, with this downregulation persisting through day 7 (Fig. 2B). Interestingly, CPSF6, a known co-factor of NUDT21 ^20^, was also downregulated in bleomycin-treated lungs (Fig. 2B), possibly due to its stability depending on its interaction with NUDT21. Similarly, NUDT21 was significantly downregulated in the lungs of LPS-treated mice compared to controls (Fig. 2E). To investigate whether this downregulation occurs in immune cells, we examined NUDT21 levels in bronchoalveolar lavage (BAL) cells from bleomycin-treated mice. NUDT21 expression was reduced in BAL cells (Fig. 2C). Since macrophages are the predominant cell type in BAL fluid during bleomycin-induced lung injury (day 3)^29^, these findings suggest that NUDT21 is downregulated in macrophages in this model. In contrast, because neutrophils are the major cells in the BAL population in LPS-induced lung injury^30^, we specifically isolated primary lung macrophages from control or LPS-treated mice using the STEMCELL F4/80 Positive Selection Kit to assess NUDT21 expression. Notably, NUDT21 protein levels were also decreased in macrophages from LPS-treated mice (Fig. 2F). Overall, these results demonstrate that NUDT21 is downregulated in macrophages in murine ARDS models, supporting its conserved role in ARDS pathogenesis.

### Myeloid Nudt21 depletion exaggerates lung injury in ARDS.

**Figure 3.**
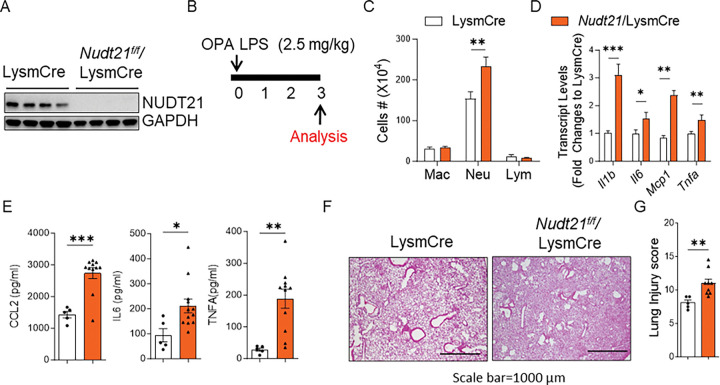
Myeloid specific NUDT21 knockout exaggerates LPS-induced lung injury. (A) NUDT21 protein levels were examined in bone marrow-derived macrophages (BMM) from Nudt21^f/f^ LysmCre and their age- and gender-matched LysmCre mice. (B) Nudt21^f/f^ LysmCre and their age- and gender-matched LysmCre mice were injected with 2.5 mg/kg LPS via OPA to induce ARDS. The lungs were analyzed on day 3. (C) The number of immune cells in the BAL. (D) The cytokine levels in the BAL cells were determined using RT-qPCR. (E) The protein levels of CCL2, IL6, and TNFA in BAL fluid were determined using ELISA. (F) Representative H&E stained lung images. (G) The lung injury scores were determined in a blind manner. N≥5. *p<0.05, ** P<0.01, *** P<0.001 vs control mice based on a two-tailed Student’s t-test. Scale bar =1000 μm.

Our human IHC data demonstrated that NUDT21 is downregulated in both macrophages and epithelial cells in ARDS lungs, suggesting potential roles in both cell types during ARDS pathogenesis. To investigate the cell-specific functions of NUDT21, we generated myeloid-specific NUDT21 knockout mice (Nudt21^f/f^LysmCre). Bone marrow-derived macrophages (BMMs) from these mice exhibited significantly reduced NUDT21 protein levels compared to BMMs from LysmCre control mice (Fig. 3A). To assess the impact of myeloid NUDT21 depletion on ARDS, we treated Nudt21^f/f^LysmCre and age- and gender-matched LysmCre controls with LPS (2.5 mg/kg) via oropharyngeal aspiration (OPA) and analyzed the lungs 3 days post-treatment (Fig. 3B). Nudt21^f/f^LysmCre mice displayed increased neutrophil infiltration (Fig. 3C) and elevated transcript levels of pro-inflammatory cytokines—including *Il1b, Il6, Ccl2*, and *Tnfa*—in BAL cells (Fig. 3D). Consistent with these findings, ELISA analysis confirmed increased CCL2, IL-6, and TNF-α protein levels in BAL fluid from Nudt21^f/f^LysmCre mice compared to controls (Fig. 3E). Histological analysis with H&E staining revealed more inflammatory cell infiltration in Nudt21^f/f^LysmCre lungs, and a blinded lung injury scoring confirmed significantly exacerbated lung injury (Fig. 3G) compared to LysmCre controls. Similarly, increased lung cytokin expression, inflammation, and injury were also observed in bleomycin-induced ARDS models using OPA in Nudt21^f/f^LysmCre mice (Supplementary Fig. 1).

To explore the role of NUDT21 in alveolar epithelial cells, we generated alveolar epithelial cell-specific NUDT21 knockout mice (Nudt21^f/f^ SPCCreER). Primary alveolar epithelial cells (~ 80–85% purity) isolated from tamoxifen-treated Nudt21^f/f^SPCCreER mice exhibited reduced NUDT21 protein levels (Supplementary Fig. 2A). We induced lung injury in both Nudt21^f/f^ SPCCreER and SPCCreER control mice using OPA LPS and analyzed the lungs on day 3. Interestingly, Nudt21^f/f^ SPCCreER mice showed no significant differences in BAL cell counts, cytokine levels, inflammation, or lung injury scores compared to controls (Supplementary Figs. 2B–F), suggesting that NUDT21 deletion in alveolar epithelial cells does not affect acute lung injury. Overall, these findings indicate that myeloid-specific NUDT21 depletion promotes inflammation and exacerbates lung injury in murine ARDS. Based on these results, we focused subsequent studies on elucidating NUDT21 signaling in macrophages.

### Exaggerated lung injury in myeloid Nudt21 KO mice persists despite neutrophil depletion.

**Figure 4.**
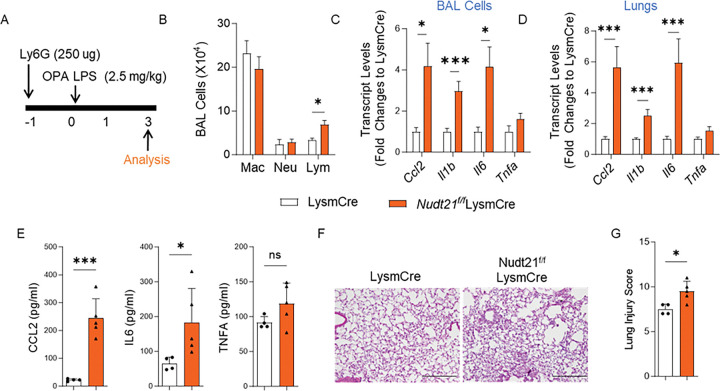
Myeloid specific NUDT21 knockout exaggerates lung injury in the absence of neutrophils. (A) Nudt21^f/f^ LysmCre and LysmCre mice were i.p. injected with 250 μg Ly6G antibody 1 day before LPS-induced ARDS. (B) The number of immune cells in the BAL. (C-D) The cytokine transcript levels in (C) the BAL cells and (D) the lungs were determined using RT-qPCR. (E) The protein levels of CCL2, IL-6, and TNF-α in the BAL fluid were determined using ELISA. (F) Representative H&E-stained lung images. (G) The lung injury scores were determined blindly. N≥4. *p<0.05, ** P<0.01, *** P<0.001 vs control mice based on a two-tailed Student’s t-test. Scale bar =1000 μm.

Since LysmCre drives gene deletion in both monocytes/macrophages and neutrophils, we sought to determine whether the exacerbated lung injury observed in Nudt21^f/f^LysmCre mice was due to NUDT21 loss in neutrophils. To address this, we depleted neutrophils in both Nudt21^f/f^LysmCre and LysmCre control mice by i.p. administering 250 μg of Ly6G antibodies one day prior to LPS exposure (Fig. 4A). Neutrophil depletion efficiency was confirmed by significantly reduced neutrophil counts in BAL fluid (Fig. 4B). Interestingly, despite effective neutrophil depletion, Nudt21^f/f^LysmCre mice still exhibited significantly elevated levels of pro-inflammatory cytokines, including *Ccl2, Il1b, and Il6* in both BAL cells (Fig. 4C) and lung homogenates (Fig. 4D) compared to LysmCre controls, indicating sustained inflammatory response even in the absence of neutrophils. Consistently, the protein levels of CCL2 and IL6 were also elevated in the BAL fluid of *Nudt21* KO mice (Fig. 4E). Histopathological examination with H&E staining revealed more severe lung inflammation and tissue damage in Nudt21^f/f^LysmCre mice compared to controls (Fig. 4F). This was further supported by blinded lung injury scoring, which showed significantly higher injury scores in Nudt21^f/f^LysmCre mice (Fig. 4G). These findings suggest that the exaggerated lung injury and inflammation observed in Nudt21^f/f^LysmCre mice are independent of neutrophils and instead point to macrophages as the key drivers of this response.

### NUDT21 depletion in CX3CR1-expressing cells exacerbates LPS-induced Lung Injury.

**Figure 5.**
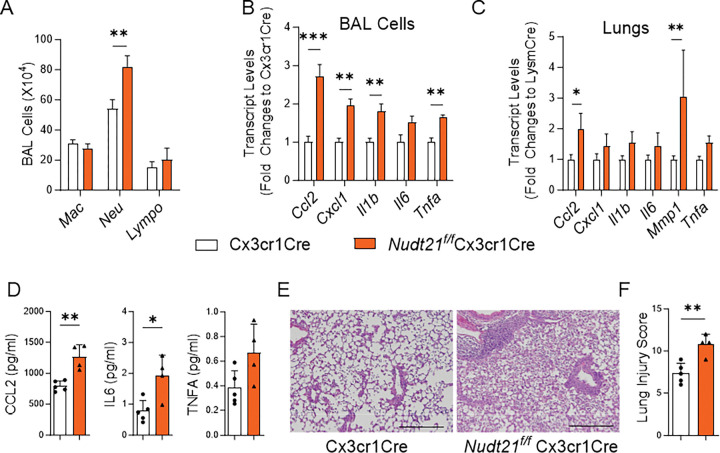
Cx3cr1Cre targeted NUDT21 deletion increases lung injury. Nudt21^f/f^Cx3cr1Cre and age- and gender-matched Cx3cr1Cre mice were injected with 2.5mg/kg bodyweight LPS, and euthanized for analysis on day 3. (A) The number of immune cells in BAL was analyzed using differential analysis. (B-C) The levels of cytokine in (B) BAL cells and (C) lungs were determined using RT-qPCR. (C) ELISA were performed to determine the cytokine protein levels in BAL fluid. (E) Representative H&E-stained lung images. Scale bar=500 μm. (F) The lung injury scores were determined in a blinded manner. N≥4. *p<0.05, ** P<0.01, *** P<0.001 vs control mice based on a two-tailed Student’s t-test.

To further confirm the macrophage-specific role of NUDT21 in ARDS and minimize the confounding effects of neutrophils, we generated Nudt21^f/f^Cx3cr1Cre mice, in which NUDT21 is selectively deleted in monocytes, macrophages, dendritic cells, and NK cells, but not in neutrophils^31, 32^. Following LPS-induced lung injury, Nudt21^f/f^Cx3cr1Cre mice showed a significant increase in neutrophil infiltration, as demonstrated by BAL differential cell counts (Fig. 5A). RT-PCR analysis revealed that pro-inflammatory cytokine levels, including *Ccl2, Cxcl1, Il1b*, *Il6*, and *Tnfa*, were significantly elevated in BAL cells from Nudt21^f/f^Cx3cr1Cre mice compared to controls (Fig. 5B). *Ccl2* and *Mmp1* were significantly upregulated in lung homogenates (Fig. 5C). Although *Cxcl1, Il1b, Il6*, and *Tnfa* did not reach statistical significance in lung homogenates, they showed a trend of upregulation in NUDT21-deficient mice. Notably, the protein levels of CCL2 and IL6 in BAL fluid were also confirmed to be increased in Nudt21^f/f^Cx3cr1Cre mice with ELISA (Fig. 5D). Consistent with these molecular findings, histological analysis with H&E staining revealed increased inflammatory cell infiltration and alveolar damage in the lungs of Nudt21^f/f^Cx3cr1Cre mice (Fig. 5E). Furthermore, blinded lung injury scoring confirmed a marked increase in lung injury severity compared to control mice (Fig. 5F). Together, these results reinforce the critical role of NUDT21 in macrophages and monocyte-derived cells in regulating inflammation and mitigating lung injury during ARDS, independent of neutrophil involvement.

### NUDT21 depletion in CD68-expressing cells does not affect LPS-induced Lung Injury.

**Figure 6.**
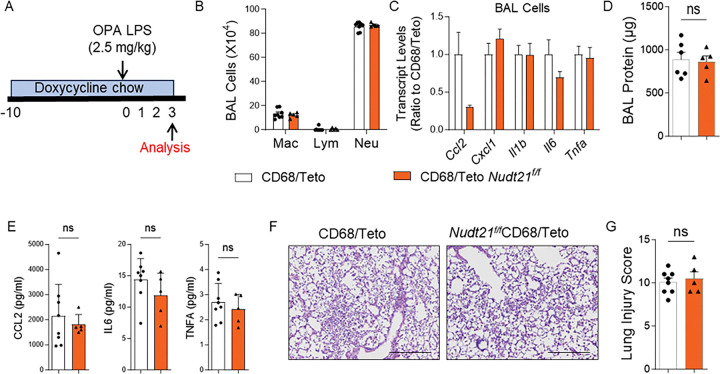
NUDT21 depletion in CD68 expressing cells does not affect LPS-induced lung injury. (A) Experimental timeline of Nudt21^f/f^ CD68rtTATetoCre and age- and gender- matched control CD68rtTATetoCre mice. (B) The number of immune cells in the BAL analyzed using differential assay. (C) RT-qPCR showed the levels of cytokine in BAL cells. (D) The total protein levels in BAL. (E) The protein levels of CCL2, IL-6, and TNF-α in BAL fluid were measured using ELISA. (F) Representative H&E-stained lung images. (G) The Lung Injury Score was assessed blindly by an expert. The lung injury scores were determined in a blinded manner. P-values were calculated based on a two-tailed Student’s t-test. N≥5. Scale bar =500 μm.

Although alveolar and interstitial macrophages both play important roles in lung homeostasis, their specific contributions to ARDS remain incompletely understood. The CD68 rtTA/tetO-Cre inducible system offers strong macrophage specificity and does not target resident alveolar macrophages, allowing for the selective study of infiltrating monocyte-derived macrophages or interstitial macrophages during ARDS^31, 33^. Here we generated Nudt21^f/f^CD68 rtTA/tetOCre mice to further validate the role of NUDT21 in macrophages. Nudt21^f/f^CD68 rtTA/tetOCre mice and control CD68 rtTA/tetO-Cre mice were fed with doxycycline chow for 10 days before inducing lung injury using oropharyngeal aspiration of LPS (Fig. 6A). Following LPS-induced lung injury, Nudt21^f/f^CD68 rtTA/tetOCre mice showed no significant differences in immune cell numbers in BAL fluid compared to control mice, as determined by BAL differential cell counts (Fig. 6B). Additionally, RT-PCR analysis revealed that key pro-inflammatory cytokines (*Ccl2, Cxcl1, Il1b, Il6*, and *Tnfa*) were not elevated in BAL cells from Nudt21^f/f^CD68 rtTA/tetOCre mice compared to controls (Fig. 6C). Histological analysis (H&E staining) and lung injury score analysis further confirmed no observable differences in inflammatory cell infiltration or alveolar damage between groups (Fig. 6D–E). Moreover, BAL protein quantification revealed no significant differences between the two groups (Fig. 6F), indicating comparable levels of lung edema. Together, these results indicate that NUDT21 deletion in CD68-expressing macrophages and monocyte-derived cells does not influence lung injury severity in the LPS-induced ARDS model, suggesting that NUDT21’s role in myeloid cells may be context-dependent and specific to certain macrophage populations.

### Modulation of NUDT21 levels in bone marrow affects the severity of ARDS.

**Figure 7.**
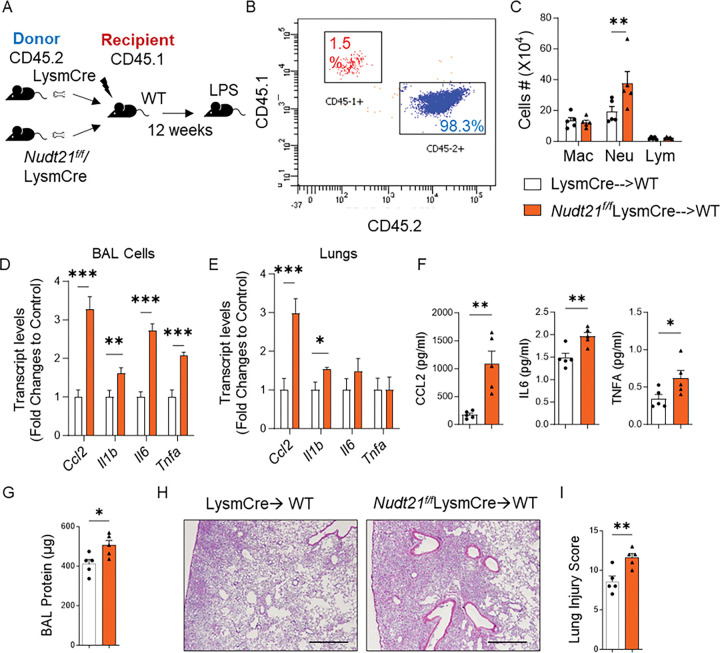
NUDT21 deletion in bone marrow promotes lung injury. (A) Schematic of the transplantation and treatment. Bone marrow from Nudt21^f/f^ LysmCre and control mice was transplanted into lethally irradiated CD45.1-bearing recipients. Bleomycin (2.5U/kg) was injected via OPA 12 weeks later to induce ARDS, and the lungs were assessed one day after. (B) Flow cytometry showing >98% of the blood cells of the recipient mice are now CD45+. (C) The number of immune cells in BAL. (D-E) RT qPCR measured transcript levels of Ccl2, Il1b, Il6, and Tnfa in (D) BAL cells and (E) Lungs. (F) The cytokine protein levels (CCL2, IL6 and TNFA) in BAL fluid. (G) Total BAL protein levels were determined using a BCA analysis. (H) Representative H&E stained lung sections. (I) Lung injury score. N=5. *p<0.05, ** P<0.01, *** P<0.001 vs control mice based on a two-tailed Student’s t-test. Scale bar =500 μm.

To further confirm the role of NUDT21 in lung injury in mice, we conducted bone marrow transplantation (BMT) experiments to determine whether changes in NUDT21 expression within hematopoietic cells influence lung injury severity. For this purpose, we transplanted bone marrow cells (BMMs) from Nudt21^f/f^LysmCre or LysmCre control mice into lethally irradiated wild-type (WT) CD45.1 recipient mice (Fig. 7A). Twelve weeks post-transplantation, flow cytometry analysis confirmed successful engraftment, with > 98% of the recipient blood cells being CD45.2-positive (Fig. 7B), indicating efficient reconstitution of the hematopoietic system with donor-derived cells. We then induced ARDS using bleomycin and assessed lung injury on day 1 post-treatment. Mice that received bone marrow from Nudt21^f/f^LysmCre donors exhibited significantly increased neutrophil infiltration in the bronchoalveolar lavage (BAL) fluid (Fig. 7C), along with enhanced vascular leakage as indicated by elevated protein levels in the BAL (Fig. 7D). Additionally, pro-inflammatory cytokines, including *Ccl2, Il1b, Il6*, and *Tnfa* were markedly elevated in the BAL fluid of these mice compared to controls (Fig. 7E). Moreover, histology and lung injury analysis showed increased inflammation and injury in the lungs of Nudt21 KO mice (Fig. 7F and 7G), highlighting an amplified inflammatory response driven by NUDT21 deficiency in hematopoietic cells.

To investigate whether restoring NUDT21 levels in bone marrow could reverse the exaggerated lung injury phenotype, we transplanted WT CD45.1 bone marrow into irradiated Nudt21^f/f^LysmCre and LysmCre control mice and subsequently induced lung injury with bleomycin. Nudt21^f/f^LysmCre mice with restored WT hematopoietic cells displayed no difference in lung inflammation and injury, neutrophil infiltration, and cytokine levels comparable to those observed in LysmCre controls transplanted with WT hematopoietic cells (Supplementary Fig. 3A–C). Collectively, these findings demonstrate that modulation of NUDT21 levels in bone marrow-derived cells can either exacerbate or alleviate lung inflammation and injury in ARDS.

### NUDT21 repression promotes proinflammatory cytokine expression through APA.

**Figure 8.**
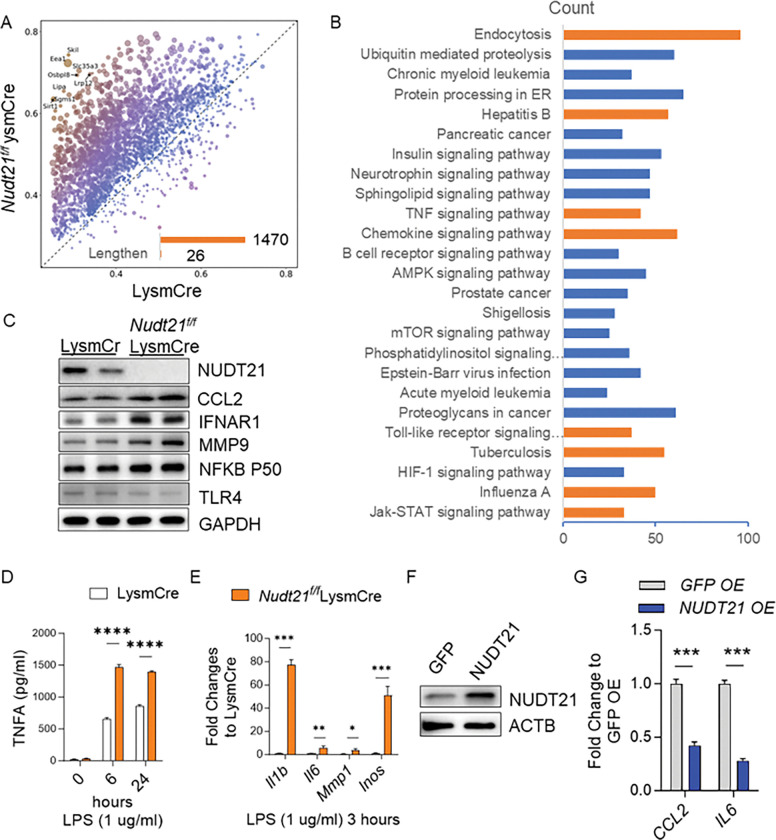
NUDT21 deletion promotes proinflammatory protein expression through APA. BMMs from LysmCre or Nudt21^f/f^ LysmCre mice were treated with LPS for 3 hours. (A) Scatterplot from RNA-Seq revealed 1470 genes had shortened 3’ UTRs. (B) Pathway enrichment of genes with 3’UTR shortening. (C) Western blot showed increased protein levels of CCL2, IFNAR1, MMP9, and NFKB1 in Nudt21 knockout BMMs. (D) ELISA measured the TFNA levels in the supernatant of cultured BMMs. (E) RT qPCR showed the transcript levels of proinflammatory cytokines, including Il1b, Il6, Mmp1, and Inos. (F-G) Thp-1-derived macrophages were transfected with GFP or NUDT21 mRNA (250 ng/ml) using using MessengerMAX^™^. (F) Western blot showed the protein levels of NUDT21 and Actb. (G) RT-qPCR analysis of CCL2 and IL6 mRNA levels in cells treated with 1 ug/ml LPS for 3 hours. N=4. *p<0.05, ** P<0.01, *** P<0.001 vs control mice based on a two-tailed student t-test.

Previous studies have demonstrated that NUDT21 downregulation induces 3’UTR shortening of target genes, thereby may enhancing their protein expression (32, 39). To investigate the molecular targets of NUDT21 downregulation in macrophages, we performed RNA sequencing to identify the APA profiles in BMMs from Nudt21^f/f^LysmCre mice compared to LysmCre controls following LPS treatment (3 hours). Our analysis identified 1,470 genes with shortened 3’UTRs and only 26 genes with lengthened 3’UTRs in NUDT21-deficient BMMs (Fig. 8A). Pathway enrichment analysis revealed that genes with 3’UTR shortening were significantly enriched in macrophage-related inflammatory pathways, including endocytosis, TNF signaling, and the chemokine signaling pathway (Fig. 7B, highlighted in orange). To determine the impact of 3’UTR shortening on protein translation, we selected six key proinflammatory genes with shortened 3’UTRs for further validation: CCL2, interferon-alpha receptor 1 (IFNAR1), matrix metalloproteinase 9 (MMP9), nuclear factor kappa B subunit 1 (NFKB1), toll-like receptor 4 (TLR4), and TNFA. Western blot analysis confirmed elevated protein levels of CCL2, IFNAR1, MMP9, and NFKB1 in NUDT21-deficient BMMs (Fig. 8C). Additionally, ELISA revealed increased TNFA secretion in the culture supernatants of NUDT21-depleted BMMs (Fig. 8D). We further assessed the mRNA expression levels of several proinflammatory cytokines and found that *Il1b, Il6, Mmp1*, and inducible nitric oxide synthase (*iNOS/Nos2*) were significantly upregulated in NUDT21-deficient BMMs (Fig. 8E). Notably, these genes did not exhibit 3’UTR shortening, suggesting that their are likely indirectly induced by other proinflammatory factors directly regulated by NUDT21, such as IFNAR1 and NFKB1.

To investigate whether restoring NUDT21 expression could suppress cytokine production, we overexpressed NUDT21 in human THP-1-derived macrophages using NUDT21 mRNA transfection via MessengerMAX^™^, a reagent known for achieving ~ 80% transfection efficiency in PBMC-derived macrophages^34^. Western blot analysis confirmed successful NUDT21 overexpression compared to control cells transfected with GFP mRNA (Fig. 8F). Upon LPS stimulation, NUDT21 overexpression was associated with a significant reduction in *CCL2* and *IL6* expression (Fig. 8G), indicating that NUDT21 negatively regulates proinflammatory cytokine production. Overall, these data provide in vitro evidence that NUDT21 deletion in macrophages promotes LPS-induced proinflammatory cytokine release through APA-mediated regulation of key inflammatory genes.

### Hypoxia suppresses NUDT21 expression through miR-181a.

**Figure 9.**
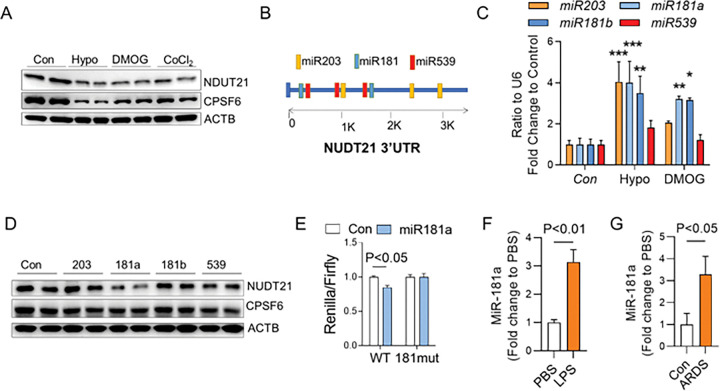
Hypoxia downregulates NUDT21 through miR-181a. Bone marrow derived macrophages (BMM) from WT mice were exposed to Hypoxia (1% O2), DMOG (1μM) or CoCl2 (100μM) for 24 hrs. (A) Western blot was performed to determine the protein levels of NUDT21, CPSF6 in BMMs. (B) Diagram showing predicted miRNA binding on NUDT21 3’UTR. (C) MiRNA expression in BMMs exposed to hypoxia or DMOG for 6 hours. (D) WT BMMs were transfected with 50 pM miRNA minics and collected for analysis after 48 hrs. NUDT21 and CPSF6 protein levels were determined by western blot. (E)The luciferase activities of WT and mutant NUDT21 (181mut, with both miR181a binding sites mutated) in 293A cells transfected with control or miR-181a mimic. (F-G) RT-qPCR shows miR-181a levels in (F) BAL cells of day 3 LPS-treated mice (1 μg/ml) and (G) trachea aspirates from control or ARDS donors. P-value were determined by ANOVA analysis followed by Dunnett multiple comparisons for C and D, and by two-tailed Student’s t-test for F and G. N≥5. * P<0.05, ** P<0.01, *** P<0.001.

Hypoxia commonly occurs in patients suffering from ARDS and hypoxia inducible factor (HIF) stabilization is observed in several murine models of ARDS in response to inflammation and injury, including ARDS induced by LPS or bleomycin^35, 36^. We next perform *in vitro* studies to demonstrate whether NUDT21 can be suppressed by hypoxia in macrophages. NUDT21 was found to be downregulated in BMMs exposed to 1% oxygen (Figure. 9A). This observation could be replicated in BMMs treated with dimethyloxalylglycine (DMOG) or CoCl_2_, two prolyl-hydroxylase inhibitors that stabilize hypoxia-inducible factors (HIF) (Figure. 9A), suggesting that hypoxia-mediated NUDT21 downregulation is through HIF stabilization. Although HIF can sometimes bind to promoters, causing repression, the more common explanation is that gene repression occurs via the induction of miRNAs. Therefore, we screened several miRNAs with at least two binding sites at NUDT21 3’UTR, including miR-203a, −181a, −181b, and – 539 (Figure. 9B). Subsequent PCR analysis indicated that miR-181a and miR-181b were consistently elevated in BMMs exposed to hypoxia or DMOG (Figure. 9C). Further studies in BMMs transfected with miRNA mimics showed that miR-181a mimics could suppress NUDT21 protein. Next to confirm the direct binding of miR-181a at *NUDT21* 3’UTR, we cloned the WT *NUDT21* 3’UTR or *NUDT21* 3’UTR with miR-181a binding sites mutation (181mut) at the 3’ end of Renilla reporter in psi-Check-2 (Promega) plasmids. Our data showed that miR-181a mimic reduced the luciferase activities of WT 3’UTR, but not the 181mut 3’UTR, suggesting that miR-181a directly binds to NUDT21 3’UTR (Figure. 9E). Consistent with these findings, miR-181a levels were increased in BAL cells of mice treated with LPS (Figure. 9F) and trachea aspirates of ARDS patients (Figure. 9G). Together, these findings suggest that NUDT21 is suppressed by hypoxia through the induction of miR-181a.

## DISCUSSION

ARDS remains a major cause of mortality in critical care, yet the molecular mechanisms that control macrophage-driven inflammation are still poorly understood. Our study identifies NUDT21 as a critical regulator of macrophage-mediated inflammation in ARDS. We demonstrate that NUDT21 is downregulated in macrophages from both human ARDS patients and murine models. Myeloid-specific NUDT21 deletion in mice resulted in increased neutrophil infiltration, vascular leakage, and elevated proinflammatory cytokines following LPS- and bleomycin-induced lung injury. Bone marrow transplantation experiments confirmed that NUDT21 loss in hematopoietic cells exacerbates ARDS severity. Mechanistically, NUDT21 deficiency induces widespread 3′UTR shortening of inflammatory genes, leading to enhanced cytokine production and tissue damage, while NUDT21 overexpression suppresses LPS-induced cytokine release. Furthermore, we uncover a hypoxia–miR-181a–NUDT21 axis that links environmental stress to APA remodeling, establishing a mechanistic connection between tissue hypoxia and macrophage inflammatory output. Together, our findings define NUDT21-mediated APA as a fundamental layer of post-transcriptional control in macrophage activation, and reveal a previously unrecognized mechanism by which hypoxia amplifies inflammation during ARDS.

Since NUDT21 exerts its function through APA regulation, understanding how APA controls gene expression in inflammatory settings is crucial. APA is a key post-transcriptional regulatory mechanism that generates mRNA isoforms with different 3’ UTR lengths, influencing mRNA stability, translation, and localization. It is controlled by a complex of cleavage and polyadenylation factors (CPAFs), including CPSF, CstF, CFI, and CFII, whose interactions shape polyadenylation site selection. APA has been implicated in various human physiological conditions and diseases immune responses^37, 38, 39^. APA also influences numerous biological and cellular processes including cell proliferation^40^, cell fate determination^41^, and senescence^42^. However, the role of APA in macrophage function and ARDS remains underexplored. Our study provides novel insights into APA regulation in ARDS, identifying NUDT21 as a key regulator of polyadenylation in macrophages. NUDT21 depletion led to widespread 3’ UTR shortening in inflammatory genes, enhancing their expression and promoting exaggerated lung injury in ARDS models. This finding aligns with previous reports on CstF-64-mediated APA regulation in macrophages, where APA alterations influence cytokine production and inflammatory signaling^21^. Additionally, NOD-, LRR- and pyrin domain-containing protein 3 (NLRP3), a central regulator of inflammation, undergoes APA in macrophages, generating a short 3′-UTR isoform that lacks key regulatory elements, including TTP (tristetraprolin)- and miRNA-223-binding sites ^43, 44^. TTP is a post-transcriptional regulator that modulates inflammation by targeting AU-rich elements (AREs) in the 3’ UTR of inflammatory genes, including NLRP3. The interplay between APA and RNA-binding proteins like TTP serves as a fine-tuning mechanism for macrophage responses, ensuring precise control of inflammatory signaling. Dysregulation of these pathways may lead to excessive inflammation, contributing to ARDS and other inflammatory diseases. Furthermore, APA is implicated in macrophage lipid metabolism, as seen in the regulation of STARD1, a gene crucial for cholesterol transport and HDL formation^45^. STARD1 undergoes APA-mediated regulation, producing transcripts with distinct 3’ UTRs that influence mRNA stability and translation, thereby affecting cholesterol homeostasis in macrophages. Similar regulatory mechanisms may contribute to ARDS pathogenesis, linking metabolic dysfunction with immune activation.

The precise mechanism by which NUDT21 downregulation promotes 3′UTR shortening is not fully understood. NUDT21 is a core component of the CFIm complex, which recognizes UGUA motifs located upstream of PAS and helps determine where cleavage and polyadenylation occur. Notably, UGUA motifs are enriched at distal PAS compared to proximal ones^46^. Under normal conditions, NUDT21 binding to these distal UGUA elements promotes distal PAS selection and the generation of longer 3′UTR isoforms. When NUDT21 is depleted, this enhancer function is lost, resulting in preferential cleavage at proximal sites and widespread 3′UTR shortening. Another potential mechanism involves destabilization of the CFIm complex. Our data and other’s^26^ show that NUDT21 depletion leads to reduced levels of its binding partners CPSF6 (CFIm68) and/or CPSF7 (CFIm59), suggesting that NUDT21 is required to maintain complex stability. Disruption of CFIm may in turn impair the recruitment of other 3′-end processing factors such as CPSF or CstF^47^. Structural studies further revealed that NUDT21 dimers complexed with CFIm68 can bridge UGUA elements through RNA looping, facilitating distal PAS activation by bringing distant sequences into proximity with the polyadenylation machinery^48^. In the absence of NUDT21, this looping mechanism is weakened, which may provide the proximal PAS with a kinetic and spatial advantage. Understanding these structural and biochemical principles opens new opportunities for drug discovery—for example, developing small molecules or RNA-based therapeutics that enhance NUDT21 stability or restore distal PAS recognition could represent a novel strategy to rebalance APA and dampen excessive inflammatory gene expression in ARDS and related diseases.

Macrophages are key regulators of lung injury and repair. In ARDS, alveolar macrophages initiate the inflammatory response by releasing pro-inflammatory cytokines, while infiltrating monocyte-derived macrophages exacerbate neutrophil recruitment and tissue damage. Despite efforts to develop a Cre-expressing mouse line that exclusively targets macrophages, no perfect system has been identified. To comprehensively investigate the role of NUDT21 in macrophages, we utilized three different Cre lines: LysmCre, Cx3cr1-Cre, and CD68-rtTA/tetO-Cre. LysmCre is widely used to target macrophages, but it also affects neutrophils^49^. Interestingly, even after neutrophil depletion, Nudt21^f/f^LysmCre mice exhibited exaggerated lung inflammation and injury compared to LysmCre controls, indicating that NUDT21 deficiency in macrophages, rather than neutrophils, drives lung injury. To further validate these findings, we used Cx3cr1-Cre, which primarily targets monocytes, macrophages, and dendritic cells, with minimal impact on neutrophils^31, 32^. In agreement with our previous results, Nudt21^f/f^Cx3cr1Cre mice exhibited worsened LPS-induced lung injury compared to Cx3cr1Cre controls, further confirming that macrophage-specific NUDT21 loss drives inflammation and injury. Finally, we used CD68-rtTA/tetO-Cre, which offers improved specificity for macrophages and monocyte-derived cells while having limited targeting of neutrophils and alveolar macrophages (AMs) ^31, 33^. Unlike our previous models, NUDT21 deletion in Nudt21^f/f^CD68-rtTA/tetO-Cre mice did not significantly affect lung inflammation or damage compared to CD68-rtTA/tetO controls. One reason could be that NUDT21 primarily regulates recruited/infiltrating macrophages (IMs) rather than alveolar macrophages in this model. Another possible explanation for this finding is that alveolar macrophages act as the first line of defense in the lung and play a primary role in LPS-induced lung injury in mice. Since CD68-rtTA/tetO-Cre does not efficiently target alveolar macrophages, it is likely that the lack of a phenotype in this model reflects the dominant role of alveolar macrophages over recruited macrophages in this setting. Together, these findings demonstrate a macrophage-specific role for NUDT21 in regulating lung injury and highlight the importance of selecting appropriate Cre models when studying macrophage function in inflammatory lung diseases.

Beyond ARDS, macrophages play a central role in numerous inflammatory diseases, where dysregulated activation contributes to chronic inflammation and tissue damage. APA-mediated regulation may be a key factor in shaping macrophage function across various pathological conditions. In fibrotic lung diseases such as idiopathic pulmonary fibrosis (IPF), COPD, and asthma, persistent macrophage-driven inflammation and altered cytokine signaling drive disease progression^50, 51^. Our previous findings in fibroblasts suggest that NUDT21-mediated APA influences lung fibrosis by regulating pro-fibrotic gene expression, potentially linking APA to broader fibrotic disorders^52^. Our study identifies a potential role of NUDT21-mediated APA in regulating inflammatory gene expression in macrophages. Given that NUDT21 depletion in bone marrow exacerbates lung injury, modulating NUDT21 levels in hematopoietic cells could serve as a potential therapeutic strategy. Moreover, considering APA’s broad role in immune regulation, further exploring the function of NUDT21 in other immune cells, such as lymphocytes could deepen our understanding of post-transcriptional control in immune-mediated diseases and uncover novel therapeutic targets.

We finally identified hypoxia-inducible miR-181a as an upstream regulator of NUDT21 in macrophages. Previous studies have reported seemingly contradictory roles of miR-181a in lung injury, suggesting that its effects may be highly cell type– and context-dependent. For example, miR-181a downregulation protected mice from LPS-induced acute lung injury by targeting Bcl-2, consistent with a detrimental role of miR-181a in epithelial injury^53^. Consistent with its proinflammatory potential, another study in macrophages demonstrated that miR-181a-5p is upregulated in LPS-stimulated RAW264.7 cells and directly targets SIRT1, where its inhibition reduced cytokine production, NF-κB activation, and sepsis severity in vivo^54^. In contrast, other work has shown that miR-181a/b-5p ameliorates inflammatory responses in monocrotaline-induced pulmonary arterial hypertension by targeting PTEN–pSTAT5–SOCS1 axis^55^, and that miR-181a-5p delivered by adipose-derived mesenchymal stem cell exosomes alleviates infection-induced lung injury through STAT3 signaling^56^. Our data add another layer of complexity by identifying hypoxia-induced miR-181a as an upstream repressor of NUDT21 in macrophages. Given that miR-181a is upregulated in LPS-treated and ARDS BAL cells, excessive miR-181a in macrophages may be detrimental by downregulating NUDT21, thereby promoting widespread 3′UTR shortening of inflammatory genes and exacerbating cytokine production. Together, these findings highlight that the role of miR-181a in lung injury likely depends on the balance of targeted pathways across different cell populations, with macrophage-specific effects contributing to inflammation in ARDS.

In summary, our study provides novel insights into the role of NUDT21 in ARDS and highlights APA as a critical regulatory mechanism in macrophage-mediated inflammation. Moving forward, it will be important to examine APA events in BAL cells from human ARDS patients using single-cell RNA sequencing to better understand the landscape and clinical relevance of APA dysregulation in the human disease setting. Additionally, systematic analysis of other core APA regulators—such as CPSF6, CSTF64, and PCF11—in the context of ARDS could reveal additional nodes of regulation and potential therapeutic targets. While small molecules directly targeting NUDT21 or APA pathways are currently unavailable, high-throughput screening approaches could be employed to identify compounds that promote 3’ UTR lengthening, thereby dampening excessive inflammatory responses. Such strategies could open new therapeutic avenues for ARDS and other immune-mediated diseases. Furthermore, given the broad role of macrophages in inflammatory disorders, future studies should investigate the function of NUDT21 and APA in other disease contexts, such as sepsis and acute kidney injury. A deeper understanding of cell type–specific APA regulation may ultimately lead to targeted therapies that fine-tune inflammatory signaling without broadly suppressing the immune system.

## METHODS

### Human Samples

Lung tissue from discarded donor lungs (controls) and patients with ARDS (PaO_2_/FiO_2_ < 350 mmHg) was obtained from the UTHealth Houston Pulmonary Center of Excellence. Human tissue collection was conducted under IRB-approved protocols (HSC-MS-15–1049 and HSC-MS-08–0354) in compliance with the Declaration of Helsinki. ARDS lung samples were collected during explant surgery and processed on-site within 60 minutes. Control lung tissue, free of acute or chronic pulmonary disease, was procured from LifeGift (Houston, TX). Comprehensive demographic and clinical information for both groups is available in Supplementary Table 1.

### Mouse Generation and Treatment

All mice utilized in this study were of C57BL/6J genetic background. Mice were maintained under pathogen-free conditions at the University of Texas Health McGovern Medical School in Houston, TX, with all experimental procedures receiving prior approval from the University of Texas Health Animal Welfare Committee (AWC 23 – 0014). Wild-type (WT), LysmCre (B6.129P2-Lyz2^tm1(cre)Ifo^/J, stain # 004781), Cx3cr1Cre (B6J.B6N(Cg)-Cx3cr1^tm1.1(cre)Jung^/J, stain # 025524), CD68-rtTA (B6.Cg-Tg(CD68-rtTA2S*M2)3Mpil/Mmjax, stain # 032044), TetoCre (b6.cg-tg(teto-cre)1jaw/j, stain #006234), SPC-CreER (B6.129S-Sftpctm1(cre/ERT2)Blh/J, stain # 028054), and CD45.1 (C57BL/6J-Ptprc^em6Lutzy^/J, strain # 033076) mice were purchased from the Jackson Laboratory. NUDT21 floxed allele mice (Nudt21^f/f^, also named CFIm25^f/f^) were generated by deleting the exons 2 and 3 of the NUDT21 gene^25, 52^ (Ozgene). Macrophage NUDT21 knockout mice (Nudt21^f/f^LysmCre, Nudt21^f/f^Cx3cr1Cre and Nudt21^f/f^CD68 rtTA/tetOCre) were generated by crossing Nudt21^f/f^ mice with LysmCre, Cx3cr1Cre or CD68-rtTA/TetoCre mice. To induce NUDT21 deletion in CD68 expressing cells, Nudt21^f/f^CD68 rtTA/tetOCre or control CD68 rtTA/tetOCre mice were fed doxycycline chow (Envigo/Inotiv, containing 625 mg/kg doxycycline in chow) starting 10 days before inducing lung injury and last throughout the experiment). Alveolar epithelial specific NUDT21 knockout mice (Nudt21^f/f^ SPCCreER) were generated by crossing Nudt21^f/f^ mice with SPCCreER mice. To induce Cre recombination, Nudt21^f/f^ SPCCreER, and age- and gender-matched SPCCreER mice were intraperitoneally (i.p.) injected with tamoxifen (37.5 mg/kg) daily for 3 days. And lung injury was induced one week after the initial tamoxifen injection.

For LPS induced lung injury model, mice were administrated with 2.5 mg/kg bodyweight LPS (Millipore Sigma, ) through oropharyngeal aspiration (OPA) and analyzed on day 1 or day 3. For the bleomycin induced lung injury model, mice were treated with 2.5 U/kg bodyweight bleomycin via oropharyngeal aspiration and euthanized on day 1 or day 3 for analysis.

For bone marrow transplantation, recipient mice were provided with neomycin sulfate (0.2%) in their drinking water starting two days prior to transplantation. On the day of transplantation, recipient mice were exposed to two doses of 500–525 RAD, administered 3 hours apart. Bone marrow cells were harvested from donor mice by flushing the femurs and tibias with sterile PBS, followed by filtration through a sterile 40 μm cell strainer to obtain a single-cell suspension. Each irradiated recipient mouse received 100 μL of bone marrow suspension (~ 5 million bone marrow cells) via retro-orbital injection under anesthesia. Post-transplantation, mice were housed in autoclaved cages with 0.2% neomycin sulfate-supplemented drinking water for 2 weeks to prevent infections during the engraftment period.

### Macrophage Isolation

Human lung tissues were cut into 1–2 mm fragments and digested in 1.68 mg/mL collagenase (Worthington Biochemical Corporation) and 50 U/mL dispase (Corning) for 30 minutes ^57, 58^. The resulting single-cell suspension was filtered sequentially through 100 μm and 40 μm strainers to remove undigested tissue. To enrich macrophages, cells were plated on IgG-coated petri dishes (prepared by incubating plates overnight with 3 mg/mL mouse IgG and washing before use) and incubated for 40 minutes to allow macrophage attachment. Non-adherent cells were removed by washing, and the attached macrophages were collected for downstream analysis.

For mouse lung macrophage isolation, lung tissues were harvested, minced, and digested using a mixture of collagenase/hyaluronidase (STEMCELL) and DNase I in RPMI 1640 medium at 37°C for 20 minutes. The digested tissue was filtered through a 70 μm nylon mesh strainer to obtain a single-cell suspension, followed by red blood cell lysis with EasySep^™^ Red Blood Cell Lysis Buffer. After washing, cells were resuspended in EasySep^™^ buffer at a concentration of 5 × 10^7^ cells/mL. For F4/80-positive selection, rat serum was added to the cell suspension, followed by the freshly prepared selection cocktail (Components A and B at a 1:2 ratio). After incubation, RapidSpheres^™^ were added, and the sample was placed in a magnetic rack for separation. The isolated F4/80⁺ macrophages were washed and collected after three rounds of magnetic separation. Purified macrophages were resuspended in RPMI 1640 medium supplemented with 10% fetal bovine serum (FBS) for downstream applications.

### Bone Marrow-Derived Macrophage (BMM) Culture

L-929 conditioned medium (LCM) was used as a source of macrophage colony-stimulating factor (M-CSF). L-929 cells (ATCC no. CCL-1) were cultured in Dulbecco’s Modified Eagle Medium (DMEM) (Thermo Fisher Scientific) supplemented with 10% fetal bovine serum (FBS) (Gendepot), 2% Glutamine (Thermo Fisher Scientific) and 1% antibiotics (Thermo Fisher Scientific) until confluence. The conditioned medium was harvested, centrifuged to remove cellular debris, and stored at − 80°C until use. BMM culture medium consisted of DMEM supplemented with antibiotics, 10% FBS, and 20% L929 conditioned medium.

For BMM isolation, mice were euthanized, and femurs were collected under aseptic conditions in sterile PBS. Bone marrow was flushed from the femurs using Dulbecco’s phosphate-buffered saline (PBS) without calcium and magnesium and passed through a 70-μm cell strainer to obtain a single-cell suspension. The cells were centrifuged at 1,200 rpm for 5 minutes at room temperature, and the pellet was resuspended in BMM culture medium. Cells were plated in non–tissue culture-treated Petri dishes and incubated at 37°C with 5% CO_2_ for 7 days to allow differentiation into macrophages. After differentiation, BMMs were detached by gentle scraping, centrifuged, and resuspended in DMEM. Macrophages were either used immediately for analysis or replated for LPS (1 ug/ml) treatments.

### Cellular Differential Assay

Mouse lungs were lavaged with 0.4 ml sterile PBS three times to collect bronchoalveolar lavage fluid (BAL). Total cell numbers in the BAL were determined using a hemocytometer. For the differential assay, BAL cells were spun onto microscope slides at 1200 rpm for 5 mins, and stained with Diff-Quick (Dade Behring, Deerfield, IL) to identify immune cell types in the BAL. The percentages of macrophages, lymphocytes, and neutrophils were assessed for the stained slides, and the number of each cell type was calculated by multiplying their percentage by the total BAL cell numbers.

### RNA sequencing and data analysis

Total RNA was extracted using the RNeasy Kit (Qiagen). RNA sequencing was performed by Dr. Eric Wagner’s group in the Department of Biochemistry and Biophysics at the University of Rochester using a novel Poly(A)-ClickSeq method that specifically targets 3΄-end mRNA sequencing^59^. Briefly, Poly(A)-ClickSeq libraries were generated from 125 ng to 2 μg of total RNA. Reverse transcription was performed using Superscript III Reverse Transcriptase (Invitrogen) with a 3΄Illumina_4N_21T primer and a mixture of AzNTP/dNTP to incorporate azido-modified nucleotide at the end of cDNA. Click-Adapter was attached to the azido-terminated cDNA via a copper-catalyzed alkyne-azide cycloaddition (CuAAC) reaction. The modified cDNA was then purified, concentrated, and amplified by PCR to create an indexed cDNA library. The library was subjected to quality control on Agilent 2200 TapeStation and to analysis on Illumina NovaSeq 6000 at Genomics Research Center at the University of Rochester. The raw data were deposited in GEO with ID GSE292996.

RNA-seq data were analyzed by Dr. Hari Krishna Yalamanchili’s group. For each sample, raw sequencing reads (PAC-Seq data) was obtained in compressed FASTQ format. PCR duplicates were identified using Unique Molecular Identifier (UMI) barcodes. UMI tools^60^ were used to extract UMI sequences from each read and append them to the read names, enabling downstream deduplication. Initial quality control is performed using fastp^61^. Adapter contamination (AGATCGGAAGAGC) is filtered using the -a option. First four nucleotides and the reads shorter than 40 nucleotides are filtered using the *-f* and *-l* options respectively. We downloaded raw FASTA sequences and annotations of the mouse genome build GRCm38 V23 from the GENCODE data portal, and aligned trimmed reads to the reference genome with Bowtie 2 ^62^ with parameters: -D 20 R 3 N 0 L 20 -i S,1,0.50 (very-sensitive-local). We indexed the reference genome using bowtie2-build default settings and saved sample-wise alignments as Sequence Alignment Map (SAM) files. We then used SAMtools^63^ V0.1.19 ‘view’, ‘sort’ and ‘index’ modules to convert SAM files to Binary Alignment Maps (BAM), coordinate sort, and index. Mapped reads are deduplicated based on the UMI marked read names and the mapping co-ordinates. After pre-processing alternative polyadenylation analysis is performed using PolyA-miner ^64^ V1.2 with the following parameters: *-pa_p 0.6 -pa_a 5 -pa_m 3 -ip 30 -expNovel 1 -t DM*. Annotated mouse polyadenylation sites from PolyA_DB3^65^ are used as reference. As the annotations were in the mm9 coordinate system, we converted them to the GRCm38(mm10) coordinate system with the liftOver program from the UCSC genome browser portal. We finally performed principle component analyses (PCA) on the PolyA cleavage site counts and confirmed clear genotype-level separation.

### Real-time Quantitative PCR

Total RNA was extracted using the Qiagen RNeasy Kit, with an additional genomic DNA removal step included during extraction. Purified RNA was then reverse transcribed using Reverse Transcription Supermix (Bio-Rad). Real-time PCR was performed on a BioRad CFX384 PCR Detection System using primers listed in Supplementary Table 2. Gene expression was quantified using the relative Ct method and normalized to β-actin or 18S rRNA, with results presented as the mean ratio.

For miRNA quantification, total RNA was reverse-transcribed using the miScript II RT Kit (Qiagen). qRT-PCR was then carried out with miScript Primer Assays (Qiagen) and the miScript SYBR Green PCR Kit (Qiagen) according to the manufacturer’s instructions. miRNA expression levels were normalized to the small nuclear RNA U6.

### Enzyme-Linked Immunosorbent Assay (ELISA)

BAL fluids were collected as described and centrifuged at 3000 rpm for 5 minutes at 4°C to remove cells. The supernatant was collected and stored at −80°C until analysis. IL-6 and TNFA levels in mouse BAL fluid were quantified using Mouse IL-6 and TNFA Quantikine ELISA Kit (R&D Systems, Cat# DY406 and DY410), following the manufacturer’s instructions. For ELISA, 100 μL of BAL supernatant and standards were added to pre-coated wells and incubated at room temperature for 2 hours. After washing, the biotinylated detection antibody was added, followed by incubation for 1 hour at room temperature. After additional washes, streptavidin-HRP conjugate was added, and plates were incubated for 30 minutes. Following a final wash, substrate solution was added for color development, and the reaction was stopped using a stop solution. Optical density was measured at 450 nm using a microplate reader. IL-6 and TNFA concentrations were calculated based on a standard curve, and results were expressed as pg/mL of BAL fluid.

### Lung histology and acute lung injury Scoring

Lung tissues were fixed in formalin, dehydrated through a graded ethanol series, paraffin-embedded, and sectioned at 4 μm thickness using a Leica RM2235 manual microtome. Tissue sections were stained with hematoxylin and eosin (H&E) and evaluated for acute lung injury by a trained investigator in a blinded manner, ensuring the analyzer was unaware of the treatment groups. Lung injury scoring was performed using a modified version of a previously established method, with damage graded as follows: 0 = no injury, 1 = injury affecting up to 25% of the tissue, 2 = up to 50%, 3 = up to 75%, and 4 = diffuse injury. Four specific injury parameters were assessed per slide: (1) atelectasis, (2) cellular infiltration, (3) alveolar wall thickening, and (4) perivascular edema. The total lung injury score was calculated as the sum of the individual parameter scores.

### Statistics

Statistical analyses were performed according to data distribution. For normally distributed datasets, comparisons between two groups were conducted using a two-tailed Student t-test. When multiple comparisons were made within an experiment, p-values from t-tests were adjusted using either the Bonferroni correction or false discovery rate (FDR) adjustment. When normality assumptions were not met, nonparametric methods were used to calculate p-values. Data are presented as Mean ± Mean Squared Error (MSE), with p < 0.05 considered statistically significant.

## Supplementary Material

Supplementary Files

This is a list of supplementary files associated with this preprint. Click to download.

• SupplementaryFigures.docx

## Figures and Tables

**Table 1.** 

Figures 1A and 1B				
Samples	Disease	Age	Gender	Race
Donor 1	Normal	37	F	Caucasian
Donor 2	Normal	32	M	Caucasian
Donor 3	Normal	56	M	Hispanic
Donor 4	Normal	59	M	Hispanic
Donor 5	Normal	44	F	Asian
Donor 6	Normal	42	F	Hispanic
Donor 7	ARDS	52	M	Hispanic
Donor 8	ARDS	68	M	Hispanic
Donor 9	ARDS	32	F	Asian
Donor 10	ARDS	39	F	Hispanic
Donor 11	ARDS	30	M	Caucasian
Donor 12	ARDS	43	F	Caucasian
Figure 1B				
Samples	Disease	Age	Gender	Race
Donor 1	Normal	29	M	Hispanic
Donor 2	Normal	62	F	Caucasian

**Table 2.** RT qPCR primers

Gene	Forward primer	Reverse Primer
Mus_Homo_18S	GTAACCCGTTGAACCCCATT	CCATCCAATCGGTAGTAGCG
Mus_Nudt21	GCGCATGAGGGAGGAATTTGA	TCCCCACCAGGTAATTTGAAGAA
Mus_Actb	CAGAACCTCTTAGGTGGGGTG	CCAGTTGGTAACAATGCCATGT
Mus_C0L1A1	GCTCCTCTTAGGGGCCACT	CCACGTCTCACCATTGGGG
Mus_FN1	GCTCAGCAAATCGTGCAGC	CTAGGTAGGTCCGTTCCCACT
Mus_Ccl2	TTAAAAACCTGGATCGGAACCAA	GCATTAGCTTCAGATTTACGGGT
Mus_Cxcl1	CTGCACCCAAACCGAAGTC	AGCTTCAGGGTCAAGGCAAG
Mus_ll1b	TGTGGCAGCTACCTGTGTCT	TCATCTCGGAGCCTGTAGTG
Mus_ll6	TAGTCCTTCCTACCCCAATTTCC	TTGGTCCTTAGCCACTCCTTC
Mus_ll8/Cxcl15	TCGAGACCATTTACTGCAACAG	CATTGCCGGTGGAAATTCCTT
Mus_Tnfa	CACCACCATCAAGGACTCAA	TCCAGCCTCATTCTGAGACA
Mus_Mmp1	CTTCTTCTTGTTGAGCTGGACTC	CTGTGGAGGTCACTGTAGACT
Mus_lnos	GTTCTCAGCCCAACAATACAAGA	GTGGACGGGTCGATGTCAC

## Data Availability

All data supporting the findings of this study are available within the main text, figures, and supplementary materials. RNA sequencing datasets have been deposited in the National Center for Biotechnology Information (NCBI) Gene Expression Omnibus (GEO) database under the accession code GSE292996. Further details are available upon reasonable request from the corresponding author.
